# Data transparency and reproducibility in health research: bridging the gap for early-career researchers

**DOI:** 10.3389/frabi.2025.1562002

**Published:** 2025-04-29

**Authors:** Sanjib Bhakta, Jianping Xie, Federico Pea, Stephen H. Gillespie

**Affiliations:** ^1^ Institute of Structural and Molecular Biology, School of Natural Sciences, Birkbeck, University of London, London, United Kingdom; ^2^ Institute of Modern Biopharmaceuticals, School of Life Sciences, Southwest University, Chongqing, China; ^3^ Federico Pea, Department of Medical and Surgical Sciences, Alma Mater Studiorum, University of Bologna, Bologna, Italy; ^4^ Stephen H. Gillespie, Division of Infection and Global Health, University of St Andrews, St Andrews, United Kingdom

**Keywords:** early-career researcher (ECR), data transparency, reproducibility, career, research culture change, health and disease

## Abstract

In the evolving and interdisciplinary landscape of health and disease research, data transparency and reproducibility are increasingly recognised as essential for maintaining scientific integrity and trust. This article invites early-career researchers (ECRs) to engage critically with these principles while navigating the pressures of academic progression, publication demands, and career development. It examines the challenges ECRs face in maintaining reproducible practices and underscores the need for institutional support, inclusive training, and mentorship across all stages of the research career. Drawing on global initiatives and case studies, the article advocates for a more collaborative, diverse, and mentally healthy research culture. It also highlights alternative career pathways beyond academia, empowering ECRs to explore opportunities in industry, government, and non-governmental organisations. By integrating transparency with values such as team science, responsible metrics, and researcher wellbeing, this article reflects the ethos of the new generation of scientists and offers a timely call for systemic change and collective action to strengthen the future of research culture.

## Introduction

In the rapidly evolving landscape of “One World, One Health” research approaches, transparency and reproducibility have become critical pillars for both established academic and industry leaders, and even more so for early-career researchers (ECRs) who work in a highly pressurised research environment. ECRs are typically in the early stages of their academic or research careers, usually within a few years of completing their doctoral studies. This group also includes individuals who may have taken career breaks, worked part-time, or shifted across disciplines. ECRs are often in postdoctoral, lectureship, or fellowship positions, where they begin leading research projects, applying for funding, and fostering collaborations for research, innovation, and knowledge exchange. Additionally, they contribute to scholarly publications, take on teaching and supervision responsibilities, and work to develop their academic and leadership profiles.

With mounting concerns over the reliability of scientific outputs and the rising number of retractions, ensuring the integrity of data has become a matter of urgency. Over the last decade, retractions in fields like life sciences saw an alarming rise (Steen et al., 2013).

The COVID-19 pandemic has notably accelerated this trend, as post-publication peer review and scrutiny of hastily published COVID-19-related studies have exposed an increasing number of untrustworthy findings (Yeo-Teh and Tang, 2022). This ongoing rise in retractions highlights the critical importance of transparency and rigorous peer-review processes (Drozdz and Ladomery, 2024) to maintain the reliability of scientific literature. At the same time, it is important to remain mindful of the growing pressure to publish and to ensure that research is timely and is part of an overall research plan. It should also be submitted to reputable, peer-reviewed journals that uphold rigorous academic standards (Elmore and Weston, 2020).

Maintaining data reproducibility while writing and publishing a scientific document is particularly challenging for ECRs, their mental health and well-being, who are at a formative stage in their careers. Professor Simon Gibbons offered a timely Frontiers in Antibiotics webinar to ECRs, “Writing and Publishing Papers – the 6Ps” and later published his expert opinion in the Journal of Natural Products (Gibbons, 2024). As the ECRs strive to establish themselves in competitive academic environments (Kent et al., 2022), mastering these aspects of research is vital for gaining credibility, fostering collaboration, and securing funding (Johnson and Weivoda, 2021).

Senior researchers often face knowledge gaps when it comes to the latest tools and methods for ensuring research reproducibility, which can hinder their ability to mentor ECRs. This gap is partly due to longstanding practices that did not emphasise the current focus on open science and rigorous data management. Many senior principal investigators may continue to rely on outdated practices that prioritise publication speed and journal impact over reproducibility and transparency (Kohrs et al., 2023). To address this issue, research institutions must ensure that training on data reproducibility is not limited to ECRs but extends across all career stages, including mid-career and senior supervisors or research (Diaba-Nuhoho and Amponsah-Offeh, 2021). Providing accessible resources and incorporating this training into mentorship programmes is crucial. Initiatives like mandatory reproducibility courses at institutions such as the NIH have made significant strides in promoting better experimental design and transparency (Devon, n.d.). By bridging this gap, institutions can help ensure that all researchers contribute to more reliable and trustworthy scientific outputs.

## Research transparency: a foundation for trust

The principle of reproducibility ensures that research findings can be replicated by independent researchers under the same conditions. This is fundamental to scientific integrity, offering a safeguard against errors, biases, and fraud. Unfortunately, the lack of transparency in research methodologies, data sharing, and documentation has led to a reproducibility crisis, with some studies proving difficult, if not impossible, to replicate (Fehr et al., 2024). Common problems in research reproducibility stem from a variety of factors. These include a lack of standardisation in methodologies, poor documentation practices, selective reporting, and challenges in replicating complex experiments under varied conditions. In many cases, researchers may omit crucial details that make replication difficult, or their data might not be as robust across different sample groups, contributing to irreproducible outcomes. Additionally, biases such as confirmation bias and the pressure to publish can also impact the integrity of research, leading to questionable reproducibility. These problems highlight the need for stronger data management protocols, open-access sharing, and collaborative platforms to ensure greater transparency and reproducibility in scientific research (Lerman et al., 2022). For example, publishing methods in journals like *Methods in Molecular Biology* provides a crucial platform to emphasise data transparency and reproducibility. By meticulously detailing experimental protocols and analyses, these publications enable researchers to replicate studies and validate findings, fostering trust in scientific advancements. Such practices align with global initiatives advocating open science, reinforcing the value of rigorous methodology in addressing irreproducibility concerns (Siegel, 2020; Batista Leite et al., 2024).

For ECRs, embracing reproducibility is not only a scientific responsibility but also a crucial pathway to establishing a strong reputation within the academic community. By using, critically analysing, and presenting data with rigour and clarity, ECRs can contribute to the integrity of research and enhance their prospects for future development. A commitment to open science practices such as data sharing and the publication of negative results fosters collaboration, avoids redundant efforts, and reduces the risk of retractions (Gorgulla et al., 2020). Moreover, transparent research practices increase visibility and allow for a broader impact across disciplines, which is invaluable for career advancement.

## Addressing the knowledge gap in supervisory support

Many senior researchers, who grew up in an era with fewer demands for research transparency, may lack expertise in the latest tools and protocols for data management and reproducibility. This presents a unique challenge for ECRs, who often rely on their supervisors for mentorship. While there is a growing recognition of the need for better training in reproducibility practices, the responsibility still largely falls on the shoulders of ECRs themselves to navigate these complex issues.

This gap underlines the need for institutions to actively support ECRs by providing training, resources, and mentorship on reproducibility and transparency. Universities must recognise the importance of this issue and integrate it into their strategic development for research and project supervision (Concordat, n.d.; Vitae, n.d.). Post-pandemic, many institutions in the UK (Birkbeck, University of London, n.d.; University College London, n.d.) have begun to shift towards open research policies, recognising the value of data sharing and reproducibility for fostering innovation and knowledge exchange.

## Promoting a collaborative and diverse research culture

In addition to transparency, a positive research culture should prioritise collaboration that recognises the value of data sharing and reproducibility and fosters innovation and knowledge exchange. ECRs often operate in isolation, concentrating on narrow aspects of their research to meet the pressing demands of publishing to secure career advancement. This compartmentalised focus, driven by the pressure to “publish or perish,” limits broader collaboration and innovation (Jamali et al., 2020). Many ECRs are caught up in fulfilling the immediate goals of producing high-quality publications, often at the expense of broader, interdisciplinary engagement. This challenge is compounded by institutional research frameworks linked to the national Research excellence framework, which emphasises publishing outcomes tied to career progression on which ECRs on short-term contacts are less dependent (Mathieson, 2017).

To support ECRs effectively, it’s essential to provide not just technical training but also collaborative opportunities and networking. Joining platforms such as the Global Reproducibility Network (UKRN) (https://www.ukrn.org/) can help ensure that ECRs are not left alone to navigate complex issues like reproducibility and open access. These networks offer resources, training, and a community for ECRs to share best practices, aligning their research with the broader goals of transparency and reproducibility. A culture of openness, where researchers feel free to share data, methodologies, and results without the fear of being scooped, is essential for driving progress in health research and beyond. Moreover, diversity in research teams, in terms of backgrounds, expertise, and approaches, can yield more comprehensive and innovative solutions to complex problems. A supportive research environment should be one that actively fosters collaboration across disciplines, encouraging ECRs to engage with experts from different fields.

## Supporting ECRs

### Beyond the research process

**Figure 1:**
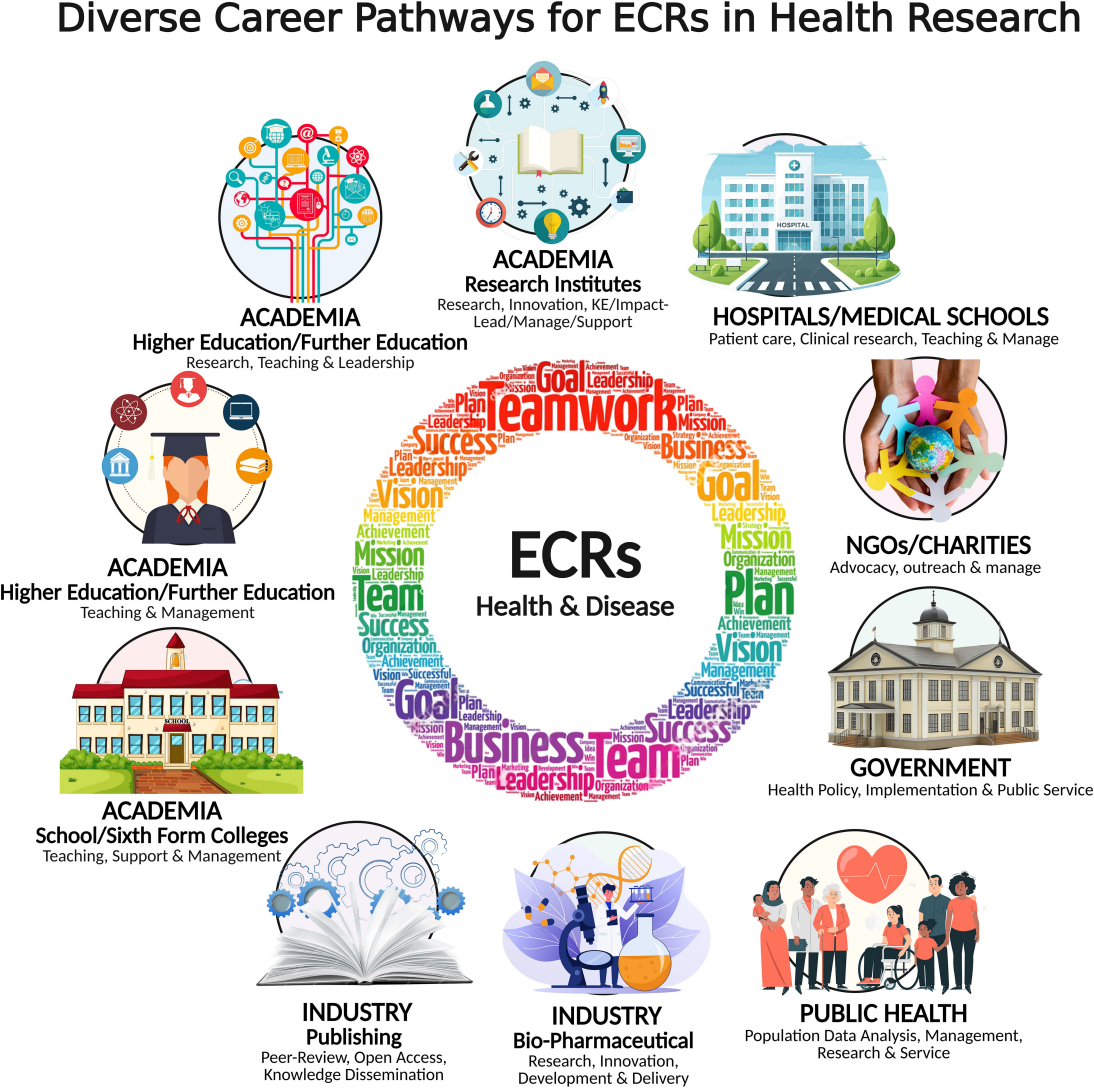
Diverse career pathways – ECRs in health research. Highlights the various roles ECRs may undertake, including postdoctoral positions, fellowships, and lectureships, and demonstrates how their career progression can span academia, industry, and other sectors. KE = Knowledge Exchange. (Collated from discussions in various ECR meetings and workshops in the UK. Created with BioRender.com, License Number: PV284AKE4D).

While mastering reproducibility is essential, ECRs also need comprehensive career support, from PhD stage to research leadership. This involves providing mentorship, professional development opportunities and skills such as oral presentations support resources. The pressures of publishing, securing funding, and navigating the academic job market can take a significant toll on researchers’ mental well-being. For example, programmes like the Wellcome Trust and Research Council UK’s pathways encourage ECRs to pursue opportunities beyond traditional academic roles, facilitating international engagements and creating networking platforms for future leadership positions. This holds true for the National Natural Science Funds of China. These initiatives aim to equip ECRs with the knowledge and confidence to pursue diverse career opportunities, whether in academia, industry, or government sectors. This year, the Biochemical Society (https://www.biochemistry.org/), together with the British Pharmacological Society (https://www.bps.ac.uk/) and the Physiological Society (https://www.physoc.org/), hosted an Early Career Symposium (Physiological Society, n.d.) led by an ECR where world-leading experts from academia, industry, and government sectors interacted closely with ECRs and shared diverse career opportunities (**Figure 1**) and discussed various aspects of research including data reproducibility.

Universities must go beyond mere rhetoric and take concrete steps to integrate mental health and well-being into their research cultures. Hosting expert panels, offering workshops on career development, and promoting open discussions about mental health are practical measures to ensure that the well-being of researchers is at the core of institutional policies. Additionally, providing access to effective data management tools and resources can help alleviate some of the pressures associated with reproducibility.

### Reimagining recognition and rewards

The traditional systems of academic recognition and rewards often prioritise individual achievements in research outputs, leaving little room to acknowledge the diverse contributions made by ECRs across education, leadership, collaboration, and societal engagement. The building of effective research networks also needs to be recognised. Good quality research and good quality teaching go hand in hand and this vital interaction should be rewarded.

To build a more inclusive and future-facing academic environment, it is essential to redefine success by embracing a broader and more balanced framework. This includes valuing the interdependence of education, research, knowledge exchange, impact, and leadership, each of which contributes to the advancement of both individual careers and the research and education sector as a whole.

Modernising recognition and rewards is not simply a matter of fairness; it is a strategic imperative. By creating flexible, diverse, and clearly defined career pathways, universities can empower ECRs to pursue roles that align with their strengths and aspirations, whether that involves innovating in teaching, leading collaborative projects, engaging with communities, or shaping institutional culture.

The Dutch Recognition & Rewards programme provides a valuable model. Its position paper (Dutch Recognition & Rewards, 2019) emphasises team science, diversity of roles, and responsible metrics as key components for evaluating academic performance. Adopting similar principles would not only support the growth and retention of talented ECRs but also cultivate a more balanced, equitable, and resilient academic ecosystem.

## Towards a manifesto for enhancing research culture

The time has come to develop an institutional manifesto that addresses the intertwined challenges of research data reproducibility and the unwavering support of ECRs. This manifesto should be a product of collaborative discussions among researchers, institutions, and policymakers, aiming to create actionable steps for fostering a positive research environment. By focusing on transparency, diversity, mental health and wellbeing, we can ensure that the next generation of researchers is well-equipped to contribute to the scientific community while thriving both professionally and personally.

As we move towards a future that must prioritise research integrity and the health & well-being subjects as well as researchers, let us commit to the principles of transparency and data reproducibility in interdisciplinary research. By leveraging the resources offered by networks such as the UK Reproducibility Network and fostering an open, collaborative culture, we can ensure that the research process becomes more transparent, reliable, and impactful. The time for change is now, and it is through collective action that we will nurture a research culture that truly supports its most valuable resource – its people.

## Data Availability

The original contributions presented in the study are included in the article/supplementary material. Further inquiries can be directed to the corresponding author.
